# The Impact of a Recent Concussion on College-Aged Individuals with Co-Occurring Anxiety: A Qualitative Investigation

**DOI:** 10.3390/ijerph20031988

**Published:** 2023-01-21

**Authors:** Jonathan Greenberg, Millan R. Kanaya, Sarah M. Bannon, Ellen McKinnon, Grant L. Iverson, Noah D. Silverberg, Robert A. Parker, Joseph T. Giacino, Gloria Y. Yeh, Ana-Maria Vranceanu

**Affiliations:** 1 Center for Health Outcomes and Interdisciplinary Research (CHOIR), Department of Psychiatry, Massachusetts General Hospital, Boston, MA 02114, USA; 2 Harvard Medical School, Boston, MA 02115, USA; 3 Dr. Robert Cantu Concussion Center, Emerson Hospital, Concord, MA 01742, USA; 4 Department of Physical Medicine and Rehabilitation, Spaulding Rehabilitation Hospital and The Schoen Adams Research Institute at Spaulding Rehabilitation, Charlestown, MA 02129, USA; 5 MassGeneral Hospital for Children Sports Concussion Program, Boston, MA 02114, USA; 6 Department of Physical Medicine and Rehabilitation, Harvard Medical School, Boston, MA 02115, USA; 7 Department of Psychology, University of British Columbia, Vancouver, BC V6T 1Z4, Canada; 8 Rehabilitation Research Program, Vancouver Coastal Health Research Institute, Vancouver, BC V5Z 1M9, Canada; 9 Biostatistics Center, Massachusetts General Hospital, Boston, MA 02114, USA; 10 Spaulding Rehabilitation Hospital, Charlestown, MA 02129, USA; 11 Department of Psychiatry, Massachusetts General Hospital, Boston, MA 02114, USA; 12 Department of Medicine, Beth Israel Deaconess Medical Center, Boston, MA 02215, USA

**Keywords:** concussion, anxiety, college-age, qualitative methods, thematic analysis

## Abstract

College-aged individuals with anxiety are vulnerable to developing persistent concussion symptoms, yet evidence-based treatments for this population are limited. Understanding these individuals’ perspectives is critical for developing effective interventions. We conducted qualitative interviews with 17 college-aged individuals (18–24 years old) with a recent (≤10 weeks) concussion and at least mild anxiety (≥5 on the GAD-7 questionnaire) to understand the life impact of their concussion. We identified 5 themes: (1) disruption to daily activities (e.g., reduced participation in hobbies and physical activity); (2) disruption to relationships (e.g., reduced social engagement, feeling dismissed by others, stigma, and interpersonal friction); (3) disruptions in school/work (e.g., challenges participating due to light sensitivity, cognitive or sleep disturbance, and related emotional distress); (4) changes in view of the self (e.g., feeling “unlike oneself”, duller, or more irritable), and (5) finding “silver linings” after the injury (e.g., increased motivation). Concussions impact the lives of college-aged individuals with co-occurring anxiety in a broad range of domains, many of which remain largely neglected in standard concussion clinical assessment and treatment. Assessing and addressing these issues has the potential to limit the negative impact of concussion, promote recovery, and potentially help prevent persistent concussion symptoms in this at-risk population.

## 1. Introduction

A concussion is the most common form of traumatic brain injury and is often associated with broad functional impairments [1,2]. Although most improve spontaneously within weeks, recovery trajectories vary, and a sizeable percentage of individuals exhibit long-term symptoms (e.g., fatigue, difficulty with memory, headache, etc.) [3,4] which can be treatment resistant [5]. Anxiety is one of the most prominent risk factors associated with persistent post-concussion symptoms [6,7,8,9,10]. 

College-aged individuals (e.g., adults 18–24 years old) are particularly prone to concussion [11], and experience higher rates of anxiety than other age groups [12]. Young adults, further, show the most rapid increase in anxiety symptoms over time compared to other age groups, nearly doubling the rates from below 8% in 2008 to nearly 15% in 2018 [13]. Their anxiety is, in part, related to a host of stressors and life circumstances unique to this age group. These include facing identity and social role ambiguity, lack of resources, challenges of newfound independence, transitions and associated difficulties adjusting to new environments, geographical relocation, challenges of finding and sustaining romantic relationships, and increased risk behavior [14,15,16]. Higher education and the academic performance pressure pose additional stressors, fueling anxiety [16,17]. Whether anxiety is present before the concussion or triggered by it, anxiety places these individuals at increased risk for developing persistent concussion symptoms. Despite this risk, college-aged individuals often “fall through the cracks” of the health system and have decreased access to care due to factors such as not living at home, being no longer under the care of their pediatrician, and not yet having established care with an adult primary care provider [18]. Evidence-based treatments for college-aged individuals with concussion are limited [19], often overlook the key role played by anxiety, and are mostly implemented in later stages after injury, despite early intervention being a promising avenue for preventing persistent symptoms [20,21,22,23]. It is therefore essential to develop effective psychosocial interventions for this population. 

To develop psychosocial treatments aimed at preventing persistent symptoms for college-aged individuals with concussion and co-occurring anxiety, it is important to first comprehensively understand these individuals’ accounts of the impact of their injury on their lives. Prior qualitative studies focusing on the experiences of individuals with concussion highlight disruptions in cognitive, emotional, physical, and social functioning [24,25,26,27,28]. Individuals with concussion report difficulty performing daily routines and work or school-related tasks, often necessitating special accommodations [25,28], as well as changes in their view of themselves and their identity [27,29,30]. However, to date, the large majority of qualitative research aiming to understand the impact of concussion has focused on individuals who are older and have sustained their injury several months or years prior. No qualitative research, to our knowledge, has focused specifically on college-aged individuals with a recent concussion and comorbid symptoms of anxiety, despite their uniquely heightened vulnerability to developing persistent concussion symptoms. 

The aim of this study is to describe the impact of a recent (≤10 weeks) concussion on the lives of college-aged individuals with co-occurring anxiety, through the lens of their own experience. Clarifying these experiences may help inform the development of psychosocial treatments to prevent the transition from acute to persistent post-concussive symptoms. 

## 2. Materials and Methods

### 2.1. Participants and Procedures

We enrolled participants for qualitative interviews as part of a broader project developing a novel mind–body intervention for this population [19]. We recruited participants via referrals from physicians and other healthcare providers at Massachusetts General (MGH) Hospital, including the MassGeneral Hospital for Children Sports Concussion Clinic and the MGH Emergency Department, as well as specialty concussion clinics in the greater Boston area. Participants were also recruited from Mass General Brigham’s patient communication platforms (e.g., Research Invitations and Partners Rally), which share research opportunities with patients who have expressed interest in research participation. Inclusion criteria were being 18–24 years old, having sustained a concussion within the previous 10 weeks, scoring ≥ 5 on the General Anxiety Disorder-7 scale [31] (indicating at least mild anxiety), and English fluency and literacy. Participants with previous brain injuries were eligible to participate as long as they were mild and symptoms from any concussion sustained in the past 2 years did not exceed 3 months (i.e., were not considered “chronic” [31,32,33,34]). Due to the mind–body focus of the intervention under development [19], we excluded participants that participated in mind–body or cognitive behavioral therapy in the past 3 months, practiced mindfulness techniques > 45 min per week on average in the past 3 months, or had a change in psychotropic medications in the past 3 months, psychosis, bipolar disorder, active suicidal ideation, substance abuse or dependence, or pregnancy. Seventeen participants fully met criteria and were the focus of our analysis. Self-reported demographic and diagnostic information is detailed in Table 1. 

Participants verbally consented to participate and received a study fact sheet prior to the qualitative interview. The Massachusetts General Hospital Institutional Review Board approved all study procedures and determined that written informed consent was not required. Participants completed a ~45-min semi-structured qualitative interview with a psychologist over Zoom. The interview focused on participants’ experiences following their concussion and the impact of their injury on their lives. Participants were asked about their symptoms, the aspects of their lives that were affected by the injury, and how these aspects of their lives were impacted. All interviews were transcribed verbatim and de-identified by a trained staff member. 

### 2.2. Data Analysis

We utilized a hybrid deductive–inductive approach [35] informed by the Framework Method for qualitative data analysis [36]. We selected this approach due to its increased use among multidisciplinary healthcare teams and systematic procedures with clear audit trails to promote rigor and transparency [36]. Our approach was deductive in that we utilized a semi-structured interview informed by prior work [24,25,26,27,28,29,30]. Our approach was inductive in that we derived initial codes using information obtained directly from participant responses through a process of open coding. First, 4 team members (2 psychologists and 2 trained research assistants) reviewed the full transcription of 2 full interviews, and conceptualized initial open codes for the full transcripts. The team then met to discuss the codes and draft a codebook. After data collection was complete, 2 research assistants separately reviewed and coded ~30% of interview transcriptions (*n* = 5) based on the initial codebook using NVIVO 12 software. Two psychologists and three research assistants from the team then met to refine the codebook. Once the initial 5 interview transcriptions were coded and compared, the codebook was finalized. One of the coders then completed coding the rest of the interview transcriptions. The lead author then reviewed the coded data, identified prominent themes, and met with other team members to refine themes and extract representative exemplar quotes.

## 3. Results

We identified five superordinate themes with corresponding subthemes that described participants’ perceptions of the impact of concussion on their lives: disruption to daily activities, disruptions in school/work, disruptions to relationships, changes to their view of themselves, and finding a “silver lining” following their injury. Themes and corresponding subthemes are summarized in Table 2, and illustrative quotes are presented in the sections below.

### 3.1. Disruption to Daily Activities

Participants reported reducing their participation in hobbies and enjoyable activities following their concussion, largely due to their physical symptoms (e.g., light or noise sensitivity) and a general sense of feeling overwhelmed. They indicated stopping or spending less time on activities such as listening to music, playing musical instruments, watching television, and playing video games. Some participants linked these reductions in enjoyable activities to a worsening in their mood or anxiety. One participant reported, “*This is kind of anxiety-inducing to just have to stop something that I was enjoying to do*”. 

Participants also decreased their level of physical activity, often due to exacerbated symptoms following the activity. One participant shared, “*I couldn’t do any physical activity because it brought on those symptoms of a headache or dizziness*”. For some, this extended to simple movement-related tasks such as lifting small objects. One participant noted, “*If I bend down the wrong way, I might start passing out […] kind of like–oh great, this thing is on the floor*”. 

Some participants attributed their difficulties in performing daily activities to concentration and memory-related symptoms. They described difficulty with tasks involving reading due to impaired comprehension, difficulty in cooking due to forgetting ingredients, and trouble remembering the location of objects. These difficulties often exacerbated participants’ emotional distress (“*I couldn’t find the peanut butter and it became this whole thing where I was going to start crying*”). 

Some participants described anxiety as a central factor disturbing their daily activities, including fear of re-injury, which made it difficult for them to go about their normal routine such as commuting (“*I was pretty nervous around sometimes crossing the street or crosswalks […], that made it a little bit harder to manage ‘cause I do commute by public transit*”). Some further described avoiding or feeling distressed in situations or places that were similar to those in which they were injured, such as crossing the street, which interrupted their ability to get around and complete their daily tasks and responsibilities. 

### 3.2. Disruptions in School/Work 

Almost all participants described reduced functioning at work or school due to their concussion. Many participants had to take time off from school or work after their injury, and some had to leave their job or halt their academic degrees. Participants who kept working often reported needing to take frequent breaks, which reduced their work productivity. This was often driven by difficulty with prolonged work in front of screens, which elicited headaches or fatigue. This difficulty also disrupted their ability to participate in meetings, many of which were held virtually over Zoom. One participant stated: “*I work from home, and everything is digital-based so that was difficult with the screens. I was getting headaches”.* Another individual indicated: “*I couldn’t be on a computer because of the screen. It kind of caused me to just put my life on halt, at a stop, at a standstill*”.

Participants also felt that sleep disturbance and fatigue following their injury disrupted their school and work functioning. Participants described difficulty waking up for class, falling asleep at work, and being frequently tired. One participant stated, “*I couldn’t do work or do school like I normally do because I just slept all day*”. Another indicated, “*It was hard to wake up for classes and I usually am very good at waking up and I’m getting everything on schedule. I fell asleep during one of my classes so it was hard for me to function*”. 

Participants described that their cognitive symptoms also took a considerable toll on their performance at school or work. They reported difficulty with reading comprehension at work, reduced reading and thinking speed, experiencing “brain fog” during their school-related responsibilities, and restricting their work to limited intervals. One participant reported, “*I wasn’t able to concentrate at all this whole summer, I’m supposed to be studying for the MCAT right now and I wasn’t able to concentrate on it and I would do a passage 10 minutes and then I would just pass out from exhaustion*”. 

These work and school-related setbacks were significant stressors for participants. They reported challenges catching up on their work and school responsibilities, which heightened their anxiety and worsened their mood. One participant reported “*Skipping school was a huge burden for me. I was just trying to keep track of how much work I was missing […] And how many tests I had to take and everything that was piling up […] it really worried me about my grades and about taking tests and even pushing those back, it’s still hanging over me now*”. Another indicated “*I’m crying and panicking a lot when too much is on my plate right now […] I’m getting down on myself because I have six classes […] when I think about all I have to do I just get really upset*”.

### 3.3. Disruptions to Relationships

Most participants reported considerable disruptions in their interpersonal relationships due to their concussion symptoms. Many participants reduced their engagement in social activities and reported a sense of isolation. Some distanced themselves because they found social interactions exhausting (“*…it made me really tired to talk to people, so my whole social life was kind of ruined. I just couldn’t deal with talking to people*”), or out of fear of exacerbating their concussion symptoms. Others secluded themselves and described a sense of loss due to an inability to engage in activities that previously provided a social setting, such as attending school and work, participating in leisure screen-based social activities on their phone and playing video games with others. Some participants avoided social interactions out of fear of embarrassing themselves, upsetting others due to their cognitive symptoms, or because they encountered difficulty following conversations. One participant stated, “*I secluded myself… because I don’t want to make a fool of myself […] it’s just the memory portion of it like not being able to follow along with a story*”. Another shared, “*I kept on being worried that I was gonna annoy people, because I was forgetting so much*”. 

Participants often felt their injury and symptoms were being dismissed or minimized by others, leading to frustration and an increased sense of isolation. Some attributed this dismissal to a social perception that a concussion is a “minor” injury. One participant stated, “*My teachers and even family members honestly were kind of ‘well, it’s just a concussion’ and I was like no this is a serious thing […] my anxiety got worse because I was wondering if people thought I was a slacker or if I was lazy*”. Another shared “*I felt really uncomfortable sharing it with my friends and... like the people in my life […] ‘Oh you can go to school, you’re fine, you just shouldn’t hit your head again and you’re a little bit dizzy, but you’re OK*”. Others expressed feeling misunderstood by others and struggling to keep up with their expectations. One participant noted, “*Everyone else expected me to do the things I would normally do and just didn’t understand that I’m worried that I’m hurting my brain and no one understood that, ‘cause they couldn’t see it, they couldn’t see the pain that I felt*”.

Despite struggling with anxiety after their injury, many participants felt they had to keep their experiences to themselves. Some felt that sharing their feelings of anxiety would be a burden on others or alienate them. One participant indicated “*It shuts down conversation. I think it drives people away […] they’re not trying to get a double dose of problems. They want to feel good, and you know, I don’t want to meet them with that kind of sentiment*”. Other participants were hesitant to share their anxiety with others due to embarrassment and concerns about perceived stigmatization. For example, one participant said, “*I guess there’s a humiliation factor that comes with it. For me, that makes me hesitant to talk about it*”. Participants’ worries about sharing their experiences exacerbated their sense of loneliness and isolation. 

Participants also reported friction in their interpersonal relationships arising from mood changes, primarily feeling more irritable. One participant described, “*More arguments with people and stuff. And I feel bad […] I just feel awful that I’ve yelled at my girlfriend over a stupid thing […] it’s just like I get aggravated quickly […] why am I getting so angry at the people I love the most*”. 

### 3.4. Changes to Participants’ View of Themselves

The disruptions in daily activities, function at school/work, and relationships led some participants to shift their view of themselves following their injury. For many, this shift was driven by their cognitive symptoms. Participants described feeling “dumb”, “stupid”, “useless”, and a sense of losing their sharpness, intelligence, and mental agility due to their memory and concentration difficulties. One participant noted her symptoms “*Made me feel stupid, the brain fog… I just never felt so dull and I hated that*”. Another said, “*I also don’t feel as smart […] it’s definitely tough dealing with not being me*”. 

Some described a change in how they view themselves due to their irritability. One participant indicated “*I was like snapping at my mom for every little thing yelling at her and I was like I thought to myself like this isn’t me but I couldn’t change it I was just super irritable […] I was just super irritable, so just fear of like who I am and what I was like becoming”.* Another participant described changes in his mood and disposition after his concussion, saying, “*I’ve been a lot more agitated and irritated than normal. I’m pretty laid back and like...the littlest things just bug me now*”. Others described a more general sense of “not being themselves” or being “detached” from themselves. This was a significant source of anxiety, prompting fears they are “stuck like this forever” or will “never feel normal again”. 

### 3.5. Finding a “Silver Lining”

Two participants expressed finding a “silver lining” following their concussion. They indicated that their concussion or anxiety had motivated them to increase their efforts or dedication to their work and school responsibilities. One participant noted, “*It actually changed my life for the better. I actually started focusing on school and trying to be better […] actually changed my life for good I wasn’t focused on school but after that I like I said I felt really stupid and so then I started focusing on school and I’ve been focused on school ever since. It’s provided me like this sustained passion to pursue academics, which is awesome*”. Another indicated about his anxiety–“*It kind of helped me be more motivated, and, like, try harder*”. 

## 4. Discussion

College-aged individuals with a recent concussion and co-occurring anxiety face unique challenges and are at particularly high risk of transitioning from subacute to persistent concussion symptoms. This study presents the first qualitative investigation of the impact of concussion on the lives of this population. Using a hybrid deductive-inductive approach, we identified five themes characterizing the impact of concussion on the lives of these individuals: disruption to daily activities, disruptions in school/work, disruption to relationships, changes in participants’ view of themselves, and finding “silver linings” after their injury.

Participants described an impairment in numerous aspects of their daily activities following their injury, including reducing their engagement in hobbies and enjoyable activities, reducing their physical activity and everyday movement, and an impaired ability to perform tasks such as reading or cooking. They attributed these changes in functioning to a perceived exacerbation of physical symptoms or anxiety about such exacerbation, concentration and memory difficulties, and fear of re-injury. These findings support previous work highlighting the impact of concussion on daily activities [24,37]. They are, further, in line with the fear-avoidance model, which underscores the interactions between pain/symptoms after injury, anxiety, avoidance of activity, and perpetuation of symptoms and disability [38,39]. The fear-avoidance model received ample support in chronic pain, as well as growing support in concussion [10,40,41,42]. This model may provide a useful lens for treatment programs to view returning to activity after concussion. While rest and significant activity restriction is advisable in the days immediately following concussion, mounting evidence suggests that prolonged activity avoidance can delay recovery and exacerbate disability [43,44]. Treatment programs (e.g., within the context of rehabilitation or psychotherapy) for this population may benefit from correcting misconceptions about prolonged rest and avoidance being conducive to recovery and emphasizing the importance of controlled and gradual return to daily and physical activities, even in the face of concussion symptoms. Including mind–body and cognitive skills (e.g., acceptance and reframing) may further aid individuals in re-engaging in activities [45]. 

Participants experienced marked disruptions in their academic and school work, including decreased productivity, having to take time off, taking frequent breaks, or quitting their studies or work. Participants related these challenges to exacerbated symptoms due to light or screen sensitivity, sleep disruptions, and cognitive symptoms such as impaired concentration and memory. These disruptions amplified participants’ anxiety and emotional distress, which supports previous findings indicating impaired academic and work performance following concussion [25,46,47]. These disruptions also provide context on how these difficulties relate to the physical, cognitive, and emotional experiences of these individuals. Despite the unique academic needs and challenges associated with concussions sustained among the college-aged population, staff members at the office for disability services in colleges have limited knowledge about students’ needs after concussion, and often perceive them as indistinguishable from those of students with other disabilities (e.g., learning disabilities and ADHD) [48]. Treatment programs for this population may benefit from encouragement to work closely with their supervisors, professors, and offices for disability to convey their needs and adopt a proactive approach for self-advocacy to facilitate adaptive return to academic and professional activities [25]. Social support and motivation have also been associated with a successful return to work after brain injury [49], and may be targeted and strengthened within the context of concussion treatment. 

Participants experienced marked disruptions in their relationships following their injury. These were characterized by social withdrawal and isolation, driven in part by the restriction of daily activities described above in social settings, and fear of symptom exacerbation. Participants felt misunderstood and dismissed by others, in part due to their injury not being visible. They were largely hesitant or embarrassed to share their experiences with others, expressed concern about mental health stigmatization, and experienced interpersonal friction due to irritability. These findings support previously reported studies regarding social disruptions following concussion [24,27,50], yet provide a novel emphasis on the central role played by anxiety in limiting social engagement and soliciting social support in this population. Despite accumulating evidence that interpersonal factors are important determinants of individual health and recovery from injury, social aspects of recovery often receive little or no attention in common treatments for concussion [50]. Incorporating a socially-informed approach to the treatment of concussion, including addressing the interplay between concussion symptoms, anxiety, and social withdrawal, might facilitate recovery. Such a socially-informed approach may be of particular importance for college-age individuals given the unique social challenges, social role ambiguity, and heightened anxiety characterizing emerging adulthood [12,15].

Participants described a sense of “not being themselves”. Some viewed themselves as “dull”, “stupid,” or less mentally capable compared to before their injury due to their cognitive symptoms. Others described a change in their temper, becoming more irritable and agitated. This change in participants’ experience of themselves caused distress and exacerbated worries that they would “never feel normal again”. Previous research highlights a sense of “loss of self” [29] and “biographical disruption” [30] following concussion, resulting in identity ambiguity. The relatively brief period after injury in the current study (3–10 weeks) may be too short to conclude the effects of people’s concussions on their true sense of identity. Nevertheless, these findings provide novel evidence that changes in how individuals view themselves can occur early after injury. This finding may provide an opportunity for early psychosocial intervention to help maintain a cohesive self-narrative. Further, these findings suggest that treatment programs may benefit from providing education on the common transience of these symptoms, which may potentially help decrease changes in identity if and when symptoms become more persistent [27,29,30].

A minority of participants expressed finding a “silver lining” following their injury. This “silver lining” manifested primarily in the invigoration of motivation, passion, or interest to try harder and succeed in their life endeavors. This finding supports prior evidence of post-traumatic growth following brain injury [51]. A recent meta-synthesis highlighted the framing of traumatic brain injury as a “new beginning” promoting a renewed desire to pursue goals as a strategy promoting resilience after moderate–severe traumatic brain injury [52]. Our results extend these findings, indicating that such renewed desire is evident after mild (rather than only moderate or severe) traumatic brain injury, and in earlier stages after injury than previously found. Treatments implemented early after concussion may aid patients in finding meaning and renewed purpose after their injury as a tool to promote resilience and recovery [53]. 

In addition to informing treatment programs, the findings of this study may help broaden and optimize the selection of assessment measures for recovery after concussion that are personalized for the situations and experiences of individual patients. These may include measures that capture disruption and avoidance of daily activities after traumatic brain injury (e.g., the Fear-Avoidance Behavior after Traumatic Brain Injury Questionnaire; FAB-TBI [41]), measures of disruptions in relationships (e.g., Interpersonal Support Evaluation List (ISEL)) [54] and PROMIS measures of social isolation [55] and emotional support [56]), measures related to individuals’ view of themselves (e.g., the Self-concept and Identity Measure (SCIM) [57]), and measures of positive psychological growth after TBI (e.g., Positive Changes in Outlook Questionnaire (CiOP) [58]). Assessing domains these individuals feel are most bothersome and impactful on their lives may help conceptualize and evaluate recovery in a way that better aligns with their lived experience. 

### Study Limitations

Some limitations of the current study should be considered. Our sample consisted of college-aged individuals (18–24 years old) with at least mild symptoms of anxiety. While targeting this group is important for the development of tailored interventions for this unique population, it is possible that the findings are unique to this population and do not reflect the experiences of other individuals with concussions. The relatively limited racial and ethnic diversity in this sample should also be noted. Further, given that participants sustained a recent concussion and experienced co-occurring anxiety, it may be difficult to differentiate the specific impact of the concussion from that of anxiety, particularly when interviewing at a single point in time. Anxiety may have been present prior to the concussions or triggered by it. A longitudinal examination of these individuals may provide a deeper understanding of the interplay between symptoms of anxiety and concussion. Given the considerable overlap in symptoms, devising psychosocial treatments based on these individuals’ experiences and challenges may benefit them above and beyond specific etiology. 

## 5. Conclusions

This study offered the first qualitative investigation of the impact of concussion on the lives of college-aged individuals with a recent concussion and co-occurring anxiety. Findings highlight a broad range of impacted domains, including disruption to daily activities, disruptions in school/work, disruption to relationships, changes in view of the self, and finding a “silver lining” after their injury. Including outcome measures in these domains may help assess recovery after concussion in a way that better aligns with individuals’ lived experiences and personal challenges after concussion. Treatment programs for this population may benefit from promoting a gradual return to daily and physical activities and minimizing avoidance, incorporating a systemic lens addressing interpersonal impacts of concussion, emphasizing the time-limited nature of symptoms and their amenability to treatment to minimize shifts in identity, and encouraging finding new meaning and motivational engagement to help cultivate resilience. Including such components in treatment programs has the potential to help limit the negative impact of concussion on people’s lives, promote recovery, and potentially help prevent persistent concussion symptoms. Future research can test the trajectory of people’s experience with the impact of their concussion longitudinally after addressing these factors in treatment, and may utilize assessment measures that correspond with the themes highlighted in the current work regarding people’s multifaceted experiences following their injury. 

## Figures and Tables

**Table 1:** Demographics.

Characteristics	Value
Age (years), mean (SD, range)	20.59 (1.88, 18–24)
Sex, *n* (%)	
Male	6 (35)
Female	11 (65)
Ethnicity, *n* (%)	
Hispanic or Latinx	1 (6)
Not Hispanic or Latinx	16 (94)
Race, *n* (%)	
White	14 (82)
Asian	3 (18)
Education, *n* (%)	
Less than high school (<12 years)	2 (12)
Completed high school or GED (12 years)	4 (24)
Some college/Associates Degree (<16 years)	6 (35)
Completed 4 years of college (16 years)	5 (29)
Marital Status, *n* (%)	
Single	15 (88)
Living with someone in a committed relationship	2 (12)

**Table 2:** Themes and sub-themes.

Theme	Sub-Theme
Disruption to daily activities	Reducing hobbies/enjoyable activities
	Decreased physical activity
	Impact of cognitive symptoms on daily activities
	Impact of anxiety on daily activities
Disruptions in school/work	Disruptions due to light/screen sensitivity
	Disruptions due to sleep disturbance and fatigue
	Disruptions due to cognitive symptoms
	Impact of disruptions on emotional distress
Disruptions to relationships	Difficulty in social engagement due to concussion symptoms
	Feeling dismissed or misunderstood by others
	Stigma and concerns about disclosure
	Interpersonal friction
Changes in view of the self	Changes related to cognitive symptoms
	Changes related to irritability
Finding a “silver lining” following the injury	

## Data Availability

The data presented in this study are available on request from the corresponding author.
